# Omitting axillary dissection in breast cancer patients with one to two positive sentinel nodes undergoing mastectomy: a retrospective and prospective cohort study with a target trial emulation

**DOI:** 10.1097/JS9.0000000000004708

**Published:** 2026-04-15

**Authors:** Haitong Xie, Xin Wang, Xianlin Gu, Chihua Wu, Wanying Guo, Rui Li, Binxu Qiu, Zhihao Zhang, Peng Gao, Xiangyue Meng, Liang Du, Shengchun Liu, Michael Gnant, Jie Chen

**Affiliations:** a Department of General Surgery, West China Hospital, Sichuan University, Chengdu, Sichuan, China; b Breast Center, West China Hospital, Sichuan University, Chengdu, Sichuan, China; c Chinese Evidence-Based Medicine Center, West China Hospital, Sichuan University, Chengdu, People’s Republic of China; d Innovation Institute for Integration of Medicine and Engineering, West China Hospital, Sichuan University, Chengdu, People’s Republic of China; e Department of Breast Surgery, Sichuan Provincial People’s Hospital, School of Medicine, University of Electronic Science and Technology of China, Chengdu, China; f Department of Breast Surgery, The First Affiliated Hospital, and College of Clinical Medicine of Henan University of Science and Technology, Luoyang, China; g Ningxia Hui Autonomous Region People’s Hospital, Yinchuan, China; h Department of Breast and Thyroid Surgery, The First Affiliated Hospital of Chongqing Medical University, Chongqing, China; i Comprehensive Cancer Center, Medical University of Vienna, Vienna, Austria; j Austrian Breast & Colorectal Cancer Study Group, Vienna, Austria

**Keywords:** axillary lymph node dissection, breast cancer, sentinel lymph node biopsy, targeted trial emulation

## Abstract

**Background:**

Previous trials have demonstrated the safety of omitting axillary lymph node dissection (ALND) in cT1-3, cN0 breast cancer patients with one to two sentinel lymph node (SLN) involvement undergoing breast-conserving surgery (BCS). However, their applicability to mastectomy patients – who do not routinely receive chest wall irradiation – remains unclear. This study aimed to evaluate whether omitting ALND is noninferior to completion of ALND among patients undergoing mastectomy.

**Methods:**

We emulated a target multicenter, nonblinded, noninferiority trial to compare sentinel lymph node biopsy only (SLNB only) and SLNB plus ALND using combined prospective and retrospective cohorts. Eligible patients had cT1-3, cN0 breast cancer with one to two SLN metastases. The primary endpoint was 5-year recurrence-free survival (RFS). Noninferiority was defined as a hazard ratio (HR) of less than 1.54 comparing the SLNB-only group to the ALND group. This study was prospectively registered with Chictr.org.cn.

**Results:**

Among 1090 included patients, 438 (40.2%) received SLNB only and 652 (59.8%) received ALND. After a median follow-up of 4.74 years, 88 patients experienced recurrence or died. The estimated 5-year RFS was 91.5% (95% CI, 89.1–93.9) in the SLNB only group and 91.8% (95% CI, 88.7–95.1) in the ALND group (HR = 0.83; 95% CI, 0.51–1.33; P_noninferiority_ = 0.005). Primary findings were consistent across subgroups and sensitivity analyses.

**Conclusion:**

Results of this targeted trial revealed that ALND can be omitted in patients undergoing mastectomy with clinically node-negative, cT1-3 invasive breast cancer who had one to two SLN metastases. These findings further inform the de-escalation of axillary surgery in breast cancer management.

## Introduction

Axillary lymph node dissection (ALND) has long been considered the gold standard for axillary staging among patients with breast cancer. However, in the mid-1990s, sentinel lymph node biopsy (SLNB) emerged as a less invasive alternative for patients with clinically node-negative breast cancer^[^1,2^]^. In the past decade, randomized trials have demonstrated that omitting ALND and performing SLNB only instead yield outcomes comparable to those of more extensive surgery even in patients with one or two positive sentinel lymph nodes (SLN)^[^3–5^]^.

These trials, however, primarily included patients undergoing breast-conserving surgery (BCS), for whom adjuvant radiotherapy (RT) is standard, and contributes to axillary disease control^[^6–8^]^. Since RT is not routinely administered post-mastectomy patients. Axillary management strategies in patients undergoing mastectomy with one to two positive SLNs remains highly debated. Moreover, these trials primarily enrolled older individuals with limited nodal diseases, a group likely to exhibit a lower risk of axillary recurrence^[^3,4,9^]^. Therefore, there remains a knowledge gap regarding optimal axillary management strategies for patients undergoing mastectomy with limited nodal involvement.

We investigated whether omission of ALND would not worsen survival outcomes, using the target trial emulation approach to avoid key biases in observational studies^[^10^]^.

## Methods

### Study design

This study aimed to emulate a target pragmatic multicenter, noninferiority trial involving both the retrospective and prospective cohorts with a conserved clinical noninferiority margin of 5%. The emulation approach is described by Hernan and Robins^[^11^]^. This study has been reported in accordance with the Strengthening the Reporting of Cohort Studies in Surgery (STROCSS) criteria^[^12^]^.

### Patient selection

We collected data from electronic health records from nine cancer centers in China from 1 September 2010 to 30 September 2022. Furthermore, since 1 October 2022, we have conducted a prospective cohort study in which eligible patients were assigned to the SLNB-only group or the ALND group. Institutional review board approval was obtained at each site. Patients provided written informed consent before their data were used in the study. The principal investigator at each site was responsible for data collection and transfer, while data cleaning and analysis were performed centrally. The study was authorized by the Ethics Committee of Registering Clinical Trials and was prospectively registered at Chictr.org.cn.

Adult female patients (aged at least 18 years) with cT1-3 N0 invasive breast cancer treated by mastectomy who had a maximum of two SLN metastases were eligible for selection in this emulated trial. To emulate the screening and randomization process of an RCT and to mitigate the risk of immortal time bias, we enrolled only patients who underwent both ALND and SLNB during the same operation based on the results of intraoperative frozen sections of SLNs (Table 1). Only patients receiving preoperative axillary ultrasonography examinations were included. cN0 status was defined by the absence of abnormal lymph node imaging. In cases of suspicious but nonpalpable lymph nodes (iN+), a negative pathology from core biopsy or a fine needle aspiration biopsy was required. SLNs with micrometastases (≤2 mm in the largest dimension) or macrometastases (>2 mm in the largest dimension) in frozen sections or final paraffin sections were defined as positive. Additionally, SLNs with isolated tumor cells were not regarded as positive nodes. The exclusion criteria were as follows: T4 tumor; extra axillary or distant metastasis (DM); a history of invasive breast cancer, bilateral breast cancer, neoadjuvant systemic therapy, a positive margin after mastectomy, or previous therapy of the ipsilateral axilla, or no radiation records, or discordant findings between intraoperative frozen section and final paraffin histopathology. Due to the time span of patient inclusion, all tumors were restaged based on the eighth version of the *AJCC Cancer Staging Manual*^[^13^]^.

**Table 1:** Summary of key elements of target trial and emulation trial components.

	Description under target trial	Description under emulation
Eligibility criteria	Female patients with cT1-3 N0 invasive breast cancer undergoing mastectomy who had 1-2 SLN metastases,	Female patients with cT1-3 N0 invasive breast cancer undergoing mastectomy who had 1-2 SLN metastases between 2012 and 2024 as recorded in the Electronic Medical Record Database, with eligibility determined solely by baseline values and not by any subsequent data.
Treatment strategies	Mastectomy and SLNB are primary therapy with ALND or no further treatment based on intraoperative frozen SLNB pathology either during the primary surgery or within 3 months later	Mastectomy and SLNB are primary therapy with ALND or no further treatment based on intraoperative frozen SLNB pathology during the same surgery; those who received ALND during a second surgery will be excluded
Assignment procedures	Patients will be randomly assigned to undergo ALND or no further axillary treatment based on the pathology result of SLNB; investigators and patients are aware of the assignment strategies	Patients will be assigned to undergo ALND or no further axillary treatment based on their observed treatment. To account for differences in baseline characteristics and confounding by indication, a propensity score-weighted cohort will be constructed.
Follow-up period	Follow-up starts at randomization and ends at relapse or recurrence, death, loss to follow-up, or administrative end of follow-up (31 December 2024), whichever occurs first	Follow-up starts at primary surgery and ends at relapse or recurrence, death, loss to follow-up, or administrative end of follow-up (31 December 2024), whichever occurs first to avoid immortal time bias
Outcomes	Primary outcomes:	Same as target trial
	5-year recurrence-free survival (RFS)	
	Secondary outcomes:	
	5-year overall survival (OS); 10-year RFS; 10-year OS; rate of any invasive recurrence, including local recurrence, regional recurrence, and distant metastasis	
Causal contrast of interest	Intention-to-treat effect; per-protocol effect	Observational analogue of the per-protocol effect
Statistical analysis	Effect estimates for the 5-year RFS and OS will be derived using a Cox proportional hazards regression model, with adjustments for baseline and postbaseline covariates, and censoring applied at deviations from the protocol. A one-sided non-inferiority test will be conducted. The rate of any invasive recurrence – including local, regional, and distant recurrences – will be compared between groups using a competing risks model, accounting for the potential impact of competing events on the outcomes.	Same as the target trial, except that inverse-probability weighting was used to account for the selection bias.

ALND, axillary lymph node dissection; CI, confidence interval; EMR, electronic medical record; IQR, interquartile range; OS, overall survival; RFS, recurrence-free survival; SLN, sentinel lymph node; SLNB, sentinel lymph node biopsy.

HIGHLIGHTS

- Very limited data exist on axillary lymph node dissection (ALND) omission in mastectomy patients.
- Sentinel lymph node biopsy only is feasible and noninferior to completing ALND in mastectomy patients with limited sentinel lymph node involvement.
- This is the largest study evaluating ALND omission in mastectomy patients with one to two SLN metastases.
- The oncologic safety of ALND omission remains consistent regardless of whether patients received adjuvant radiotherapy.

### Procedures

The SLNB procedure was conducted with a dual tracer combined with blue dye and technetium Tc 99 m. Local treatment of the breast included mastectomy with or without irradiation of the chest wall. For the SLNB process, the removal of at least three SLNs is recommended. ALND requires the dissection of at least anatomical levels I and II, including at least 10 lymph nodes. Palpable abnormal lymph nodes that were removed during surgery were not classified as SLNs.

If systemic therapy was indicated, it was administered after completing the axillary surgery. Adjuvant systemic therapy and radiotherapy were administered in accordance with NCCN guidelines. Axillary radiotherapy was recommended for patients who underwent mastectomy when at least four lymph node metastases were detected after ALND. To date, no standardized radiotherapy guidelines exist for mastectomy patients without axillary dissection. Axillary radiotherapy involved all three levels of the axilla and supraclavicular fossa. The prescribed dose was 25 fractions of 2 Gy. During follow-up, a physical examination was conducted every 6 months for the first 3 years, followed by annual examinations thereafter. Annual mammography was mandatory, and other examinations were performed based on to the patients’ symptoms.

### Outcomes

The primary endpoint was 5-year recurrence-free survival (RFS), defined as the time from surgery to RFS events. RFS events include invasive recurrence and death, based on the updated Standardized Definitions for Efficacy End Points (STEEP) criteria^[^14^]^. The secondary endpoints were 5-year overall survival (OS), 10-year RFS, 10-year OS, local recurrence (LR), regional recurrence (RR), and DM rates. RR indicated recurrence in the ipsilateral axilla, the supra- or infraclavicular region, and the internal/intra-mammary lymph nodes. LR included recurrence on the chest wall, and DM included recurrence in other locations. If locoregional regional and DM occurred in one patient, the calculation of the LR, RR, and DM rates was conducted separately. Invasive recurrence rates refer to events occurring from the date of surgery to the last follow-up assessment. Overall survival was defined as the interval from the date of surgery to the date of death for any reason. Patients were censored if they were lost to follow-up, died due to a reason unrelated to breast cancer, or were still alive at the 5-year or 10-year follow-up without any events.

Events were centralized across participating centers using a prespecified case-report form. Participating center investigators abstracted events from imaging, pathology, and clinical notes. Assessors were not blinded to surgical procedure, but event definitions were objective and uniformly applied across all centers.

### Statistical analysis

Descriptive clinicopathological data were summarized for two groups using the Mann‒Whitney U test or t test for continuous variables, while categorical data were compared using the Fisher exact test or chi-square test. Kaplan‒Meier curves were used to evaluate 5-year RFS and 5-year OS. The effect of omitting ALND on 5-year RFS was assessed by a Cox proportional hazards model. Hazard ratios (HRs; SLNB only vs ALND) are presented with 95% CIs. Cumulative incidence functions (CIFs) were estimated using the method of Fine and Gray, which accounts for competing risks. Between-group comparisons were performed using Gray’s test, a weighted log-rank test for CIFs^[^15^]^. In the estimation of LR, RR, DM, and death were considered competing risks, while for DM rate estimates, death was deemed a competing risk. The detailed statistical methods for handling missing data are provided in the appendix.

To mitigate the effects of selection bias and confounding factors in this nonrandomized study, we constructed propensity score models via Inverse Probability Treatment Weighting (IPTW). Propensity scores for each patient group were estimated via multivariable logistic regression, incorporating covariates including age, tumor stage, tumor histological type, number of SLN macrometastases, number of SLN micrometastases, focal invasion, lymphovascular invasion, chemotherapy status, radiotherapy status, and subtype. These covariates were selected based on clinical expertise and existing literature suggesting causal relationships between these variables and both surgical intervention and outcomes. These propensity scores were then used to calculate individual patient weights. Finally, we applied a weighted Cox proportional hazards model to estimate the 5-year RFS HR between the SLNB only and ALND groups. To assess whether the weighting procedure achieved adequate covariate balance between groups, we computed the standardized mean differences (SMDs) for all confounders; the comparisons are presented in Supplemental Digital Content eFigure 2, available at: http://links.lww.com/JS9/H284.

On the basis of literature and clinical experience, the 5-year RFS was estimated to be 90% for patients who have undergone mastectomy with ALND. A noninferiority margin of 5% was set, allowing a reduction in the 5-year RFS rate to 85% for patients treated with SLNB alone. The noninferiority margin was defined as the upper bound of the 95% confidence interval (CI) for a HR of 1.54. To achieve a power of 80% (*β* = 0.2) at a significance level of *α* = 0.1 for a noninferiority design, the required sample size was estimated to be 1078 patients (444 in the SLNB only group and 634 in the ALND group). In addition to HR, we prespecified a non-inferiority margin based on restricted mean survival time (RMST). Non-inferiority was defined as a 5-year RMST for SLNB-only that was no more than 1 month shorter than that for completion of ALND^[^16–18^]^. RMST was assessed as the between-group difference with corresponding 95% CI. Other endpoints, including 5-year OS, 5-year locoregional recurrence (for competing risk analysis, CRA), and 5-year DM (for CRA), were evaluated in a similar manner, as outlined in Supplemental Digital Content eTable 1, available at: http://links.lww.com/JS9/H284. The detailed noninferiority margin settings and sample size calculations are shown in the appendix.

We used similar methods of primary analysis to assess the impact of ALND omission on prespecified clinically relevant subgroups: age, tumor stage, tumor grade, tumor histological type, subtype, type of largest positive SLN, focal invasion, lymphovascular invasion, chemotherapy status, and radiotherapy status. Interaction terms between treatment group and each subgroup variable were included in the subgroup analyses, and *P* values for interaction were calculated. The results are presented with HRs (95% CIs) in a forest plot, unadjusted for multiplicity. Formal statistical tests were not conducted for the comparisons.

Sensitivity analyses were conducted using a model adjusted for the calendar period as a potential risk factor for nonadherence to the assigned treatment. Patients who had at least nine lymph nodes removed from the dissection group were compared with the total SLNB only group. Sensitivity analysis was then extended to exclude patients lost to follow-up within 5 years to investigate whether the effects differed for patients with a shorter follow-up duration.

We performed multivariable analysis on RFS using the proportional hazards regression model. The baseline factors that may affect the safety of omitting dissection together with the treatment group were assessed in the multivariable model. The multivariable model was examined for multicollinearity. Multivariable cox regression analysis was restricted to eligible patients with no missing data. We also conducted multivariable analysis after IPTW with the whole cohort.

Statistical analysis was performed using R, version 4.3.2 (R Core Development Team). On the basis of literature and clinical experience, a noninferiority margin of 5% was set, allowing a reduction in the 5-year RFS rate for patients with early-stage breast cancer and one to two SLN metastases treated with SLNB alone. A one-sided noninferiority test was performed by conducting a Z test on the logarithm of the HR.

## Results

**Figure 1.**
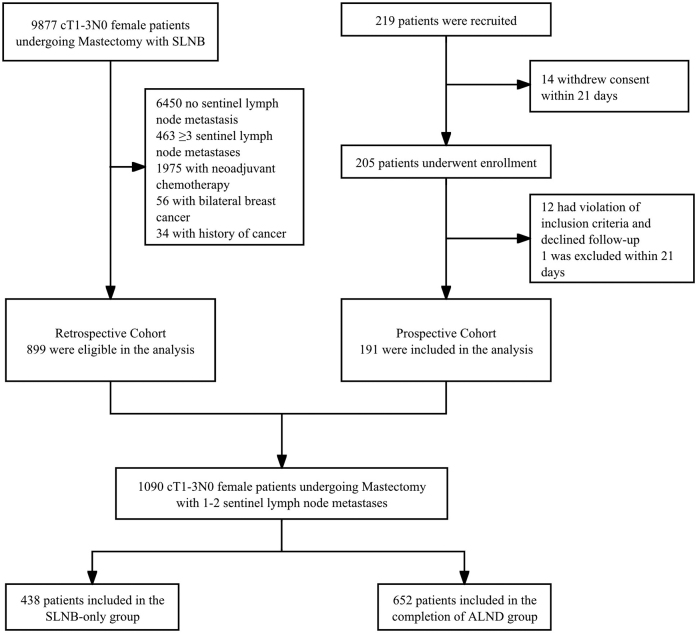
Trial profile.

We identified 9877 adult female patients with cT1-3N0 invasive breast cancer who underwent mastectomy with SLN biopsy cultures across nine hospitals in China between 2012 and 2024. A total of 3427 patients had positive SLN metastases and were eligible for our emulated trial (Fig. 1). Among the 1090 patients who had one to two SLN metastases, 438 (40.2%) patients underwent SLNB only, and 652 (59.8%) patients underwent both SLNB and ALND. Detailed information on the missing data is provided in Supplemental Digital Content eTable 2, available at: http://links.lww.com/JS9/H284.

Demographic and clinicopathological characteristics of the patients are summarized in Table 2. The median age (IQR) was 49 (43–57) years, with 550 patients (50.5%) under 50 years of age. The age distribution was similar between the groups [median: 50 years (range, 43–57; SLNB only) vs 49 years (42–57; ALND)]. Most patients in both groups had pT1-2 tumors [97.2% (426/438; SLNB only) vs 98% (639/652; ALND)], hormone receptor-positive and human epidermal growth factor receptor 2 (HER2)-negative tumors [68.7% (301/438) vs. 66.9% (436/652), *P* = 0.94], and Grade 2 tumors [67.4% (295/438) vs. 66% (430/652), *P* = 0.90]. No significant differences were found in terms of tumor pathological characteristics, including tumor type, Ki-67 score, and focal location. However, significant differences were observed in terms of lymphovascular invasion in the primary tumor [20.8% (83/438, SLNB only) vs. 14.4% (93/652, ALND), *P* = 0.009]. Conversely, patients who underwent ALND were more likely to receive radiotherapy [ALND: 78.5% (512/652) vs SLNB only: 58.7% (257/438), *P*<0.001] and chemotherapy [ALND: 96% (626/652) vs SLNB only: 93.2% (408/438), *P* = 0.048]. With respect to radiotherapy, we further detailed the treatment fields (Table 2). All RT was delivered at 50 Gy in 25 fractions. Detailed information about radiation planning approaches is not yet available. The tumor subtype distribution was well balanced between the groups, and the adjuvant chemotherapy regimens used were similar. The baseline characteristics of the two groups before and after the generation of overlap weights were balanced by using SMDs, with values of less than 0.10 for all the variables, as shown in Supplemental Digital Content eFigure 1, available at: http://links.lww.com/JS9/H284 of Supplementary. The weights generated from the propensity scores had a median of 0.34 (IQR, 0.30–0.51). SMDs < 0.10 indicated well-balanced groups, mimicking a randomized trial. The groups were well balanced in terms of baseline characteristics after IPTW (Supplemental Digital Content eTable 3, available at: http://links.lww.com/JS9/H284).

**Table 2:** Baseline patient and tumor characteristics.

	Patients, No. (%)			
Characteristic	Overall	SLNB only	Completion ALND (*N* = 652)	*P* value
	(*N* = 1090)	(*N* = 438)		
**Baseline**				
Age at surgery, y				
Mean (SD)	50.17 (10.86)	50.44 (11.19)	49.98 (10.64)	0.489
<40	171 (15.7)	68 (15.5)	103 (15.8)	0.362
40–49	379 (34.8)	147 (33.6)	232 (35.6)	
50–64	428 (39.3)	169 (38.6)	259 (39.7)	
65–74	86 (7.9)	42 (9.6)	44 (6.7)	
≥75	26 (2.4)	12 (2.7)	14 (2.1)	
Median (IQR)	49 (43–57)	50 (43–57)	49 (42–57)	0.597
Type of breast surgery				
Mastectomy	858 (81.9)	329 (82.2)	529 (81.8)	0.907
Reconstruction	189 (18.1)	71 (17.8)	118 (18.2)	
Focal				
Unifocal	971 (92.7)	363 (90.8)	608 (94)	0.067
Multifocal	76 (7.3)	37 (9.2)	39 (6.0)	
No. of removed SLNs				
1–2	196 (18.0)	52 (11.9)	144 (22.1)	<0.001
3–4	582 (53.4)	235 (53.7)	347 (53.2)	
≥5	312 (28.6)	151 (34.5)	161 (24.7)	
Mean (SD)	3.72 (1.29)	3.88 (1.20)	3.61 (1.33)	<0.001
Median (IQR)	4 (3–5)	4 (3–5)	4 (3–4)	0.001
No. of positive SLNs				
1	792 (72.7)	324 (74.0)	468 (71.8)	0.467
2	298 (27.3)	114 (26.0)	184 (28.2)	
Mean (SD)	1.27 (0.45)	1.26 (0.44)	1.28 (0.45)	0.426
Median (IQR)	1 (1–2)	1 (1–1)	1 (1–2)	0.426
No. of SLN macrometastasis				
0	77 (7.1)	49 (11.2)	28 (4.3)	<0.001
1	782 (71.7)	306 (69.9)	476(73.0)	
2	231 (21.2)	83 (18.9)	148 (22.7)	
Mean (SD)	1.14 (0.51)	1.08 (0.54)	1.18 (0.49)	<0.001
Median (IQR)	1 (1,1)	1 (1,1)	1 (1,1)	0.002
No. of positive ALNs				
Mean (SD)	0.58 (1.48)	0.02 (0.14)	0.29 (0.18)	<0.001
Median (IQR)	0 (0,1)	0 (0,0)	0 (0,1)	<0.001
Chemotherapy status				
Yes	1034 (94.9)	408 (93.2)	626 (96.0)	0.048
No	46 (5.1)	30 (6.8)	26 (4.0)	
Radiotherapy status				
cw + sc	453 (41.6)	52 (11.9)	401 (61.5)	<0.001
cw + sc + ax	319 (29.3)	209 (47.7)	110 (16.9)	
No	318 (29.2›)	177 (40.4)	141 (21.6)	
				
**Tumor characteristics**				
Pathological tumor stage				
pT1	510 (46.8)	217 (49.5)	293 (44.9)	0.298
pT2	555 (50.9)	209 (47.7)	346 (53.1)	
pT3	13 (1.2)	7 (1.6)	6 (0.9)	
Unknown	12 (1.1)	5 (1.2)	7 (1.1)	
Histological type				
Ductal	1031 (94.6)	410 (93.6)	621 (95.2)	0.572
Lobular or mixed	33 (3.0)	17 (3.9)	16 (2.5)	
Others	23 (2.1)	10 (2.3)	13 (2.0)	
Unknown	3 (0.3)	1 (0.2)	2 (0.3)	
Lymphovascular invasion				
Yes	176 (16.8)	83 (20.8)	93 (14.4)	0.009
No	871 (83.2)	317 (79.2)	554 (85.6)	
Grade				
1	38 (3.5)	16 (3.7)	22 (3.4)	0.898
2	725 (66.5)	295 (67.4)	430 (66.0)	
3	323 (29.6)	125 (28.5)	198 (30.4)	
Unknown	4 (0.4)	2 (0.5)	2 (0.3)	
Subtype				
ER+,HER2−	737 (67.6)	301 (68.7)	436 (66.9)	0.943
ER+,HER2+	132 (12.1)	53 (12.1)	79 (12.1)	
ER−,HER2+	109 (10.0)	43 (9.8)	66 (10.1)	
ER−,HER2−	109 (10.0)	40 (9.1)	69 (10.6)	
Unknown	3 (0.3)	1 (0.2)	2 (0.3)	
Ki-67 index				
<20	338 (31.0)	140 (32.0)	198 (30.4)	0.859
≥20	747 (68.5)	296 (67.6)	451 (69.2)	
Unknown	5 (0.5)	2 (0.5)	3 (0.5)	
Mean	29 (0.18)	28 (0.18)	29 (0.18)	0.405
Median (IQR)	25 (15–40)	25 (15–40)	30 (15–40)	0.259

Data are mean (SD), or median (IQR) or number (%). Tumor subtype was classified according to estrogen receptor (ER) status and human epidermal growth factor receptor 2 (HER2) amplification. HER2 positive was defined as an immunohistochemistry (IHC) score of 3+ or 2+ with confirmation of amplification by fluorescence in situ hybridization (FISH). ALND, axillary lymph node dissection; ax, axillary; cw, chest wall; EMR, electronic medical record; ER, estrogen receptor; HER2, human epidermal growth factor receptor 2; IQR, interquartile range; OS, overall survival; sc, supraclavicular; SD, standard deviation; SLN, sentinel lymph node; SLNB, sentinel lymph node biopsy.

Loss to follow-up: 2.3% (10/438) in the SLNB-only group vs 1.7% (11/652) in the ALND group at 5 years.

A median of 4 (IQR 3–5) SLNs were excised in both groups. Patients in the SLNB only group had more SLNs removed than those in the ALND group (median, 4 [IQR 3–5] vs. 4 [3–4]; *P* = 0.001). However, no significant difference was found in the median number of SLN metastases between the SLNB only group and the ALND group (median: 1 [1–1] vs. 1 [1–2]; *P* = 0.426). Most patients in both groups had macrometastatic SLNs.

In the ALND group, a median of 13 (IQR 10–16) additional lymph nodes were excised in addition to the sentinel nodes. Additional non-SLN metastases were found in 309 (47.4%) of the 652 patients after ALND. Among the patients with SLN micrometastases, five (17.9%) of the 28 patients had additional non-sentinel-lymph node metastases; among the patients with one SLN macrometastasis, 218 (45.8%) of the 476 patients had additional non-sentinel-lymph node metastases; and among the patients with two SLN macrometastases, 86 (58.1%) of the 148 had additional non-sentinel-lymph node metastases. The pathological nodal stage was pN1 (one to three metastases) in all patients in the SLNB only group and in 559 (85.7%) patients in the ALND group. The pathological nodal stage was pN2 (four to nine metastases) in 85 (13%) patients in the ALND group. The pathological nodal stage was pN3 in 10 (1.5%) patients in the ALND group.

A total of 166 (15.2%) of the 1090 patients did not receive radiotherapy, in deviation of the guidelines. Seven (1.1%) of the 652 patients who had more than four lymph node metastases after ALND did not receive radiotherapy. In the SLNB only group, 159 (36%) of the 438 patients with more than one SLN micrometastasis did not receive radiotherapy (25 had two macrometastases, 133 had one macrometastasis, and one had two micrometastases). In the ALND group, 512 (78.5%) underwent ALND combined with axillary radiotherapy. Systemic therapy adherence was greater in the ALND group [7% (30/438) in the SLNB only group vs. 5% (31/652) in the ALND group; *P* = 0.048].

**Figure 2.**
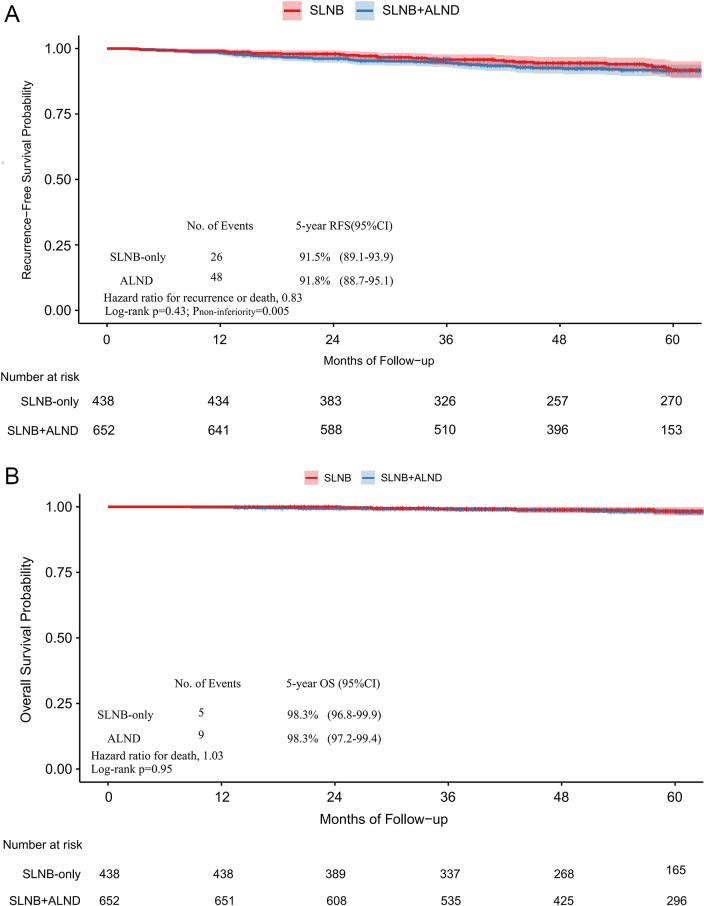
Analysis of recurrence-free survival (A) and overall survival (B) (*n* = 1090).

ALND, axillary lymph node dissection; OS, overall survival; RFS, recurrence‑free survival; SLNB, sentinel lymph node biopsy.

**Figure 3.**
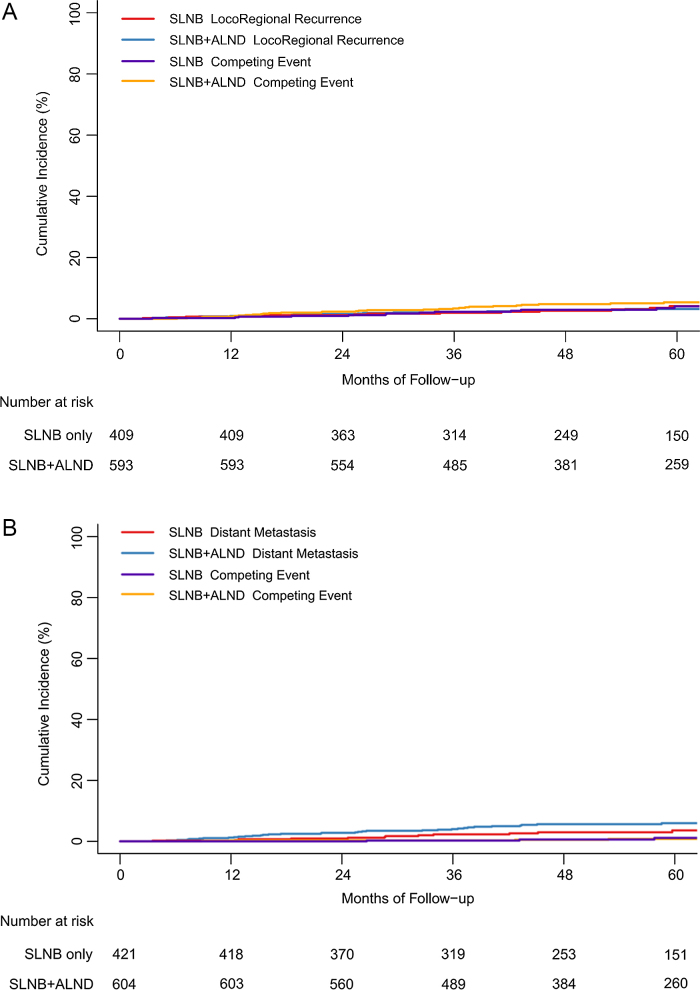
Cumulative incidence for locoregional recurrence (A) and distant metastasis (B) by group.

ALND, axillary lymph node dissection; SLNB, sentinel lymph node biopsy.

**Figure 4.**
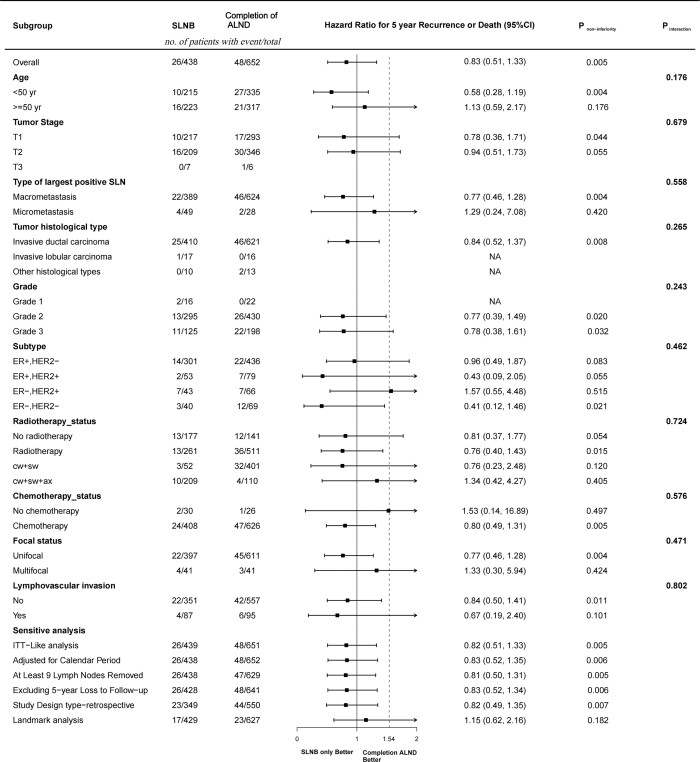
Forest plot of the subgroup analysis of 5-year recurrence-free survival.

There were no significant differences in RFS or OS between the treatment groups. The estimated 5-year RFS was 91.5% (95% CI, 89.1–93.9) in the SLNB only group and 91.8% (88.7–95.1) in the ALND group (Fig. 2). The estimated 5-year OS was 98.3% (96.8–99.9) in the SLNB only group and 98.3% (97.2–99.4) in the ALND group (Fig. 2). When evaluating the hypothesis of noninferiority for 5-year RFS, we found that performing SLNB only was noninferior to performing SLNB combined with ALND (HR, 0.83; 95% CI, 0.51–1.33; noninferiority, *P* = 0.005). Sensitivity analysis with a one-sided *α* = 0.05 confirmed that the non-inferiority conclusion remained robust. The 5-year absolute risk difference in recurrence or death was −0.4% (95% CI, −4.4% to 3.6%), indicating no statistically significant difference between groups. This finding was confirmed by sensitivity analysis (Fig. 3). We also performed a sensitivity analysis applying a more conservative non-inferiority margin of HR = 1.3. The results showed that SLNB-only was non-inferior to ALND for 5-year RFS (noninferiority, *P* = 0.031). At 5 years, the RMST difference between SLNB-only and ALND was 0.79 months (95% CI, −0.36 to 1.94; *P* = 0.18), with a one-sided 90% lower confidence limit of 0.04 months. This lower limit is well above the prespecified non-inferiority margin of −1 month, confirming that SLNB-only is non-inferior to ALND with respect to 5-year RMST. No significant difference was observed in the 10-year RFS or 10-year OS between the SLNB only and ALND groups (Supplemental Digital Content eFigure 4, available at: http://links.lww.com/JS9/H284). The subgroup analyses detected no statistically significant heterogeneity in RFS between treatments, with the observed HR below 1.54. Furthermore, subgroup analyses under the HR = 1.3 consistently supported non-inferiority across pre-specified subgroups (Supplemental Digital Content eFigure 3, available at: http://links.lww.com/JS9/H284). Interaction tests between the treatment group and baseline variables were not statistically significant (all *P* for interaction > 0.05) (Fig. 4).

Subgroups were defined based on baseline characteristics in the whole cohort (*N* = 1090). The HR for each subgroup is illustrated by a black square with the size of a square corresponding to the inverse variance of the corresponding log-HR estimate. The dashed line represents an HR of 1.54, which is the noninferiority margin for omission of axillary dissection. Sensitivity analyses were performed in a model including ITT-like analysis (evaluating patients according to their intended axillary management at surgery), adjusted for calendar year, limiting the ALND group to patients with ≥9 lymph nodes removed (compared with the full SLNB-only group), with excluding 5-year loss of follow-up, in the retrospective cohort (as the potential heterogeneity in data collection that may cause inconsistence in the primary analysis) and landmark analysis limited to cases with at least 1-year event-free follow-up to address early censoring.

The loss-to-follow-up rate was 1.93% (21 of 1090 patients) at 5 years. Detailed baseline characteristics of patients lost to follow-up are summarized in Supplemental Digital Content eTable 12, available at: http://links.lww.com/JS9/H284, and Supplemental Digital Content eTable 13, available at: http://links.lww.com/JS9/H284. A sensitivity analysis excluding patients lost to follow-up within 5 years yielded consistent results (HR = 0.83, 95% CI 0.52–1.34), confirming the robustness of the findings. At the data cutoff, the median follow-up was 4.74 years, a total of 88 RFS events occurred (29 in the SLNB only group and 59 in the ALND group; Table 3). The recurrence rate in the entire cohort was 7.2% (79/1090); the locoregional recurrence rate was 3.1% (34/1090); and the DM rate was 4.9% (53/1090). There were no significant differences in the rates of LR [1.1% (five of 438) vs.1.8% (12 of 652); *P* = 0.51] or RR [2.3% (10 of 438) vs. 1.7% (11 of 652); *P* = 0.63] between the SLNB only and ALND groups. Patients in the SLNB-only group had a lower DM rate [2.7% (12 of 438) vs. 6.3% (41 of 652); *P* = 0.01] than patients in the ALND group. Recurrence patterns were further stratified by radiotherapy status and molecular subtype in both treatment groups (Supplemental Digital Content eTables 10 and 15, available at: http://links.lww.com/JS9/H284).

**Table 3:** Recurrence-free survival analyses.

Variable	Overall	SLNB only	Completion ALND (*N* = 652)
	(*N* = 1090)	(*N* = 438)	
Recurrence-no.(%)	79 (7.2)	26 (5.9)	53 (8.1)
Local	17 (1.6)	5 (1.1)	12 (1.8)
Regional	21 (1.9)	10 (2.3)	11 (1.7)
Distant	53 (4.9)	12 (2.7)	41 (6.3)
Local only	11 (1.0)	5 (1.1)	6 (0.9)
Regional only	14 (1.3)	9 (2.1)	5 (0.8)
Distant only	44 (4.0)	11 (2.5)	33 (5.1)
Locoregional	34 (3.1)	14 (3.2)	20 (3.1)
Death-no.(%)	23 (1.7)	8 (1.8)	15 (2.3)
Breast cancer	14 (1.3)	5 (1.1)	9 (1.4)
Other causes	9 (0.8)	3 (0.7)	6 (0.9)

Local only indicates recurrence limited to the chest wall only; Regional only indicates recurrence limited to the ipsilateral axilla, the supra- or infraclavicular region, and the internal/intra mammary lymph nodes; locoregional indicates recurrence involving the chest wall and/or regional lymph nodes; distant only indicates the recurrence limited to the other.

The predictive values of the baseline variables were assessed via univariate analysis (Table 4). The factors being of clinical relevance were subsequently included in the multivariable model. The regression estimates were based on 1072 patients without missing data regarding age, tumor stage, tumor grade, tumor histological type, subtype, chemotherapy status, and radiotherapy status. No multicollinearity was detected in the multivariable regression model (Supplemental Digital Content eTable 6, available at: http://links.lww.com/JS9/H284). The non-inferiority of axillary dissection omission was consistently observed before and after IPTW. Furthermore, there were no significant interactions between these predictors and the treatment group, indicating no statistically detectable heterogeneity in the HRs across subgroups (Table 5). In the competing risk analysis, the cumulative incidences of locoregional recurrence and DM were modestly higher in the ALND group (sHR = 1.23, 95% CI 1.05–1.45; and sHR = 1.26, 95% CI 1.07–1.48, respectively) (Fig. 3).

**Table 4:** Univariate cox regression analysis for 5-year recurrence-free survival.

Variables	Unmatched		IPTW	
	Hazard ratio	*P* value	Hazard ratio	*P* value
	(95% CI)		(95% CI)	
Age	1.00(0.98,1.02)	0.8574	1.00(0.98,1.02)	0.8769
Group				
SLNB + ALND	1 (ref)		1 (ref)	
SLNB	0.83 (0.51, 1.33)	0.4302	0.82 (0.49, 1.39)	0.4570
Surgery type				
Total mastectomy	1 (ref)		1 (ref)	
Total mastectomy with reconstruction	0.70 (0.35, 1.40)	0.3134	0.86 (0.41, 1.83)	0.6992
Number of removed SLN	0.93 (0.78, 1.11)	0.4232	0.97 (0.76, 1.24)	0.8039
1 or 2	1 (ref)		1 (ref)	
3 or 4	0.60 (0.35, 1.04)	0.0704	0.55 (0.29, 1.04)	0.0665
≥5	0.71 (0.38, 1.33)	0.2852	0.8 (0.39, 1.64)	0.5417
Number of positive SLN	0.90 (0.53, 1.53)	0.6924	0.96 (0.53, 1.76)	0.9011
Number of SLN macrometastasis	0.75 (0.47, 1.21)	0.2425	0.75 (0.41, 1.35)	0.3279
Number of SLN micrometastasis	1.37 (0.74, 2.52)	0.3111	1.56 (0.8, 3.05)	0.1899
Number of ALND	1.01 (0.98, 1.04)	0.3878	1.01 (0.98, 1.04)	0.6103
Number of positive ALND	1.18 (1.08, 1.28)	0.0001 ^*^	1.19 (1.11, 1.27)	0.0000^*^
Pathological tumor stage				
pT1	1 (ref)		1 (ref)	
pT2	1.60 (1.00, 2.58)	0.0515	1.59 (0.93, 2.72)	0.0864
pT3	1.90 (0.26, 14.00)	0.5287	2.68 (0.34, 21.28)	0.3471
Focal				
Unifocal	1 (ref)		1 (ref)	
Multifocal	1.62 (0.74, 3.54)	0.2234	1.23 (0.53, 2.85)	0.6311
Lymphovascular invasion				
No	1 (ref)		1 (ref)	
Yes	1.05 (0.54, 2.04)	0.8941	0.9 (0.44, 1.84)	0.7746
Tumor histological type				
Invasive ductal carcinoma	1 (ref)		1 (ref)	
Invasive lobular carcinoma	0.48 (0.07, 3.43)	0.4626	0.63 (0.09, 4.69)	0.6512
Other histological types	1.47 (0.36, 6.01)	0.5899	1.29 (0.28, 5.88)	0.7394
Grade				
Grade 1	1 (ref)		1 (ref)	
Grade 2	1.15 (0.28, 4.77)	0.8464	0.54 (0.12, 2.53)	0.4290
Grade 3	2.24 (0.54, 9.36)	0.2670	1.11 (0.23, 5.23)	0.8947
Subtype				
ER+,HER2−	1 (ref)		1 (ref)	
ER+,HER2+	1.39 (0.67, 2.88)	0.3815	0.98 (0.45, 2.12)	0.9526
ER−,HER2+	2.74 (1.48, 5.08)	0.0014^*^	2.39 (1.22, 4.67)	0.0120 ^*^
ER−,HER2−	3.08 (1.68, 5.62)	0.0003^*^	2.65 (1.32, 5.33)	0.0070 ^*^
Ki-67	10.65 (3.55, 31.96)	0.0000^*^	7.08 (2.18, 23)	0.0015^*^
Chemotherapy status				
No Chemotherapy	1 (ref)		1 (ref)	
Chemotherapy	1.15 (0.36, 3.66)	0.8093	0.87 (0.26, 2.83)	0.8080
Radiotherapy status				
No radiotherapy	1 (ref)		1 (ref)	
Radiotherapy	0.82 (0.51, 1.33)	0.4180	0.79 (0.47, 1.33)	0.3644
Radio_cw + sc	0.96 (0.58, 1.61)	0.8863	0.92 (0.53, 1.59)	0.7603
Radio_cw + sc + ax	0.60 (0.31, 1.15)	0.1217	0.65 (0.31, 1.34)	0.2398
Number of removed LN	1.01 (0.98, 1.04)	0.4604	1.01(0.97,1.04)	0.6643

ALND, axillary lymph node dissection; ER, estrogen receptor; HER2, human epidermal growth factor receptor 2; pT, pathological tumor; SLN, sentinel lymph node; SLNB, sentinel lymph node biopsy.

**Table 5:** Multivariate cox regression analysis for 5-year recurrence-free survival.

Variables	Before IPTW		After IPTW	
	Hazard ratio	*P* value	Hazard ratio	*P* value
	(95% CI)		(95% CI)	
Group				
SLNB + ALND	1 (ref)		1 (ref)	
SLNB	0.77 (0.47, 1.26)	0.3011	0.80 (0.47, 1.37)	0.4147
Age	1.00 (0.97, 1.02)	0.6909	0.99(0.97, 1.02)	0.5207
Pathological tumor stage				
pT1	1 (ref)		1 (ref)	
pT2	1.39 (0.85, 2.25) a	0.1858	1.43(0.83, 2.48)^a^	0.1925
pT3	1.75 (0.23, 13.29)	0.5910	2.59 (0.30, 22.23)^a^	0.3794
Grade				
Grade 1	1 (ref)		1 (ref)	
Grade 2	1.21 (0.29, 5.08)^a^	0.7978	0.57 (0.11, 2.83)^a^	0.4823
Grade 3	1.72 (0.40, 7.49)^a^	0.4689	0.89 (0.18, 4.49)^a^	0.8833
Tumor histological type				
Invasive ductal carcinoma	1 (ref)		1 (ref)	
Invasive lobular carcinoma	0.61 (0.08, 4.50)^a^	0.6305	0.81 (0.10, 6.36)^a^	0.8356
Other histological types	1.67 (0.40, 6.94)^a^	0.4818	1.55 (0.31, 7.68)^a^	0.5837
Chemotherapy status				
No chemotherapy	1 (ref)		1 (ref)	
Chemotherapy	0.99 (0.28, 3.43)^a^	0.9820	0.82 (0.23, 2.98)^a^	0.7587
Radiotherapy status				
No radiotherapy	1 (ref)		1 (ref)	
Radiotherapy	0.67 (0.39, 1.14)	0.1398	0.70 (0.38, 1.30)	0.2524
Subtype				
ER+,HER2−	1 (ref)		1 (ref)	
ER+,HER2+	1.23 (0.59, 2.59)	0.5785	0.89 (0.39, 2.01)^a^	0.7739
ER−,HER2+	2.27 (1.18, 4.35)^a^	0.0137*	2.06 (1.00, 4.24)^a^	0.0504
ER−,HER2−	2.55 (1.33, 4.90)^a^	0.0049*	2.20 (1.03, 4.71)^a^	0.0421*

ALND, axillary lymph node dissection; ER, estrogen receptor; HER2, human epidermal growth factor receptor 2; pT, pathological tumor; SLN, sentinel lymph node; SLNB, sentinel lymph node biopsy.

^*^ *P*<0.05, statistically significance.

^a^ The upper bound of the 95% CI higher than the hazard ratio of 1.54.

Patients with missing information of treatment (*n* = 18) were excluded in the multivariable analysis before IPTW (*N* = 1072). After IPTW, the whole cohort was included in the multivariable analysis (*N* = 1090). All variables are from the same model. Wide CIs are due to small sample size or no recurrence events occurred.

## Discussion

Target trial emulation is being increasingly recognized as a robust methodology for observational studies, particularly in situations wherein conducting RCTs is challenging. To our knowledge, this study represents the largest combined retrospective and prospective cohort analysis assessing the real-world impact of omitting ALND on early breast cancer patients undergoing mastectomy.

Our findings demonstrated that the omission of ALND is a feasible and noninferior strategy for patients undergoing mastectomy with one to two SLN metastases, as RFS in the SLNB only group was noninferior to that in the ALND group across all clinical subgroups. Furthermore, no significant differences were observed in OS, LR, RR, or DM rates between the two treatment groups.

These results are consistent with findings from previous randomized trials, such as the SENOMAC trial^[^5^]^, which randomized 2540 cT1-3 breast cancer patients with one or two SLN macrometastases to complete ALND or its omission. After a median follow-up of 46.8 months, no differences in the endpoints were observed between the two groups; similar findings were observed among the 920 patients who underwent mastectomy. However, it has to be noted that almost 90% of patients in SENOMAC received some radiotherapy to the axilla, which is likely over-treatment. Besides, the AMAROS trial demonstrated that axillary radiotherapy is as effective as ALND in terms of achieving axillary control and survival outcomes^[^9,19^]^. Previous trials have limited applicability to real-world settings because of their focus on low-risk populations. For example, the IBCSG 23-01 trial exclusively enrolled patients with SLN micrometastases, whereas nearly 40% of patients in the ACOSOG Z0011 and AMAROS trials had this condition, which is higher than the proportion reported in real-world data. In contrast, only 7% of patients had SLN micrometastasis, thus enhancing the generalizability of our findings. Additionally, the small number of mastectomy patients in earlier trials reduces the reliability of evidence supporting ALND omission in this population.

Omission of ALND has become standard treatment in China for patients undergoing BCS with one to two positive SLNs, based on ACOSOG Z0011^[^8,20^]^ and AMAROS^[^9^]^ trials. For mastectomies, the 2017 National Comprehensive Cancer Network (NCCN) guidelines recommended omitting axillary dissection for patients who have one SLN micrometastasis^[^21^]^. However, there is still controversy over the de-escalation of ALND in patients receiving mastectomy with one to two SLN macrometastases. Mastectomy was not an eligible intervention in the ACOSOG Z0011 trial, and it was performed in only 17.4% and 9% of patients in the AMAROS and IBCSG 23-01 trials, respectively. Moreover, mastectomy remains the primary surgery type in China, accounting for approximately 80% of all breast cancer surgeries^[^22^]^. This highlights the urgent need for high-quality research specific to Chinese patients to inform axillary guidelines in mastectomy. Based on these results, our study primarily focused on the mastectomy patients with SLN macrometastases.

Retrospective analyses have evaluated the clinical outcomes of breast cancer patients with one to two SLN macrometastases who underwent mastectomy with omission of ALND^[^23,24^]^. Gao *et al.* conducted a study including 763 patients, 84 of whom received SLNB only. No significant differences in RFS and OS were observed between the SLNB-only and ALND groups, which were consistent with our findings^[^24^]^. Other ongoing prospective randomized trials are evaluating the noninferiority of ALND omission in patients with SLN macrometastases. In the SINODAR-ONE trial, patients with T1 to T2 breast cancer and sentinel-node macrometastases were randomized between the SLNB only group and the ALND group^[^25^]^. In the subgroup analysis of 218 mastectomy patients, equivalent RFS and OS rates were observed after 33 months of follow-up. The trial was later reopened to increase power in mastectomy patients. The Positive Sentinel Node (POSNOC) trial randomized 1900 patients with T1 to T2 breast cancer and one or two macrometastases to either axillary treatment (ALND or axillary radiotherapy) or no further treatment^[^26^]^.

According to the current clinical guidelines, if ALND was omitted, radiotherapy to the axilla was recommended to the breast cancer patients undergoing mastectomy with positive SLN^[^27,28^]^. Among the 159 patients in the SLNB-only group with more than one SLN micrometastasis who did not receive radiotherapy, the majority refused radiotherapy, 12 patients exhibited poor tolerance to radiotherapy, five patients had cardiovascular diseases, and 10 patients were older than 70 years old. Our study provides important complementary evidence to prior randomized trials such as SENOMAC and AMAROS, where regional nodal irradiation was administered to the nearly 90% of patients. In contrast, radiotherapy is an optional setting in our cohort that only 70.8% of patients received adjuvant radiotherapy overall. And among irradiated patients, only approximately 30% of the patients received radiation therapy to the chest wall plus regional lymph nodes. After applying IPTW, the baseline for radiotherapy was balanced, and the RFS in the SLNB-only group was non-inferior to that in the ALND group. The interaction term demonstrated that there was no statistically significant difference between the SLNB-only and ALND groups regarding the impact of radiotherapy on patient prognosis. Stratified Fine–Gray competing risk analyses showed no significant treatment × radiotherapy interactions for locoregional recurrence or DM (all *P* > 0.05), indicating that the relative treatment effect was consistent regardless of RT receipt or RT field (Supplemental Digital Content eTables 17–19, available at: http://links.lww.com/JS9/H284). The BOOG 2013-07 trial classified cT1-3 patients with one to three SLN metastases into four groups according to the axillary surgery type and radiotherapy status^[^29^]^. No significant differences in survival outcomes were observed among groups. Notably, patients without axillary treatment (neither axillary dissection nor radiotherapy) had more favorable tumor characteristics, with 75% having SLN micrometastases. In contrast, our study cohort more accurately represents the real-world settings: 41.3% of SLNB-only patients did not receive radiotherapy, and among them, 87% (158 of 177) had SLN macrometastases, whereas 23 patients had micrometastases.

RFS analyses stratified by breast cancer molecular subtype were performed (Supplemental Digital Content eTable 15, available at: http://links.lww.com/JS9/H284). Patients with ER−/HER2+ and triple-negative breast cancer exhibited relatively higher recurrence risks compared with ER+/HER2− disease. These findings suggest that biologically aggressive subtypes may be associated with greater locoregional recurrence, suggesting the axillary treatment escalation in these patients.

The de-escalation of axillary treatment for breast cancer is progressing, with recent prospective randomized trials such as the Sentinel Node vs Observation After Axillary Ultra-Sound (SOUND)^[^30^]^ and the Intergroup-Sentinel-Mamma (INSEMA) trials^[^31^]^. These trials suggest that omission of any surgical axillary staging was noninferior to SLNB for patients undergoing BCS. Although axillary surgery traditionally informed systemic therapy decisions, this role has diminished with expanding indications for adjuvant treatment. Following the NATALEE trial, ribociclib is now recommended for all node-positive HR+/HER2− early breast cancer, irrespective of nodal burden. Thus, SLNB would provide adequate information for therapy selection in current clinical practice.

The main limitation of our study was that, as an observational study, residual confounding cannot be entirely eliminated. Even though we mitigated immortal time bias by enrolling patients who underwent ALND during primary tumor surgery and conducted a target trial emulation, unmeasured factors, such as intraoperative lymph node morphology or specific surgeon preferences, could still influence the decision-making process. Additionally, the generalizability of our results may be limited. The relatively high median SLNB yield is indicative of specialized centers, and therefore, these findings should be extrapolated with caution to settings with lower surgical proficiency. Besides, given that most of the enrolled patients had luminal subtype breast cancer, which has a potential for late recurrence, the current follow-up period may still be relatively short. Furthermore, due to limited event counts in certain subgroups, statistical power to detect interactions was constrained. Despite non-significant interaction tests, numerical trends toward HR > 1 in high-risk subtypes suggest a potentially narrower safety margin for omitting ALND. These exploratory findings warrant clinical caution and require validation in larger cohorts. Future studies with longer follow-up and larger event numbers will be important to further clarify long-term recurrence risks and to confirm the durability of non-inferiority over time. In addition, to rigorously address the challenge of unmeasured confounding, future research should prioritize prospective data collection with greater granularity. This includes both structural variables, like hospital identifiers, regional radiotherapy availability, and temporal policy factors, and nuanced clinical decision factors, like intraoperative surgical assessments and patient preferences. Such comprehensive data will enable advanced causal inference approaches to further validate these findings.

The strength of this study was that our results support the real-world clinical practice of omitting axillary surgery for cT1-T3 N0 breast cancer patients who underwent mastectomy, based on a large number of well-documented patients.

## Conclusion

In general, the waiving ALND was noninferior in patients who underwent mastectomy with cT1-T3 N0 breast cancer and one to two SLN metastases. Radiotherapy does not interfere with the noninferiority effect of omitting axillary dissection; thus, SLNB likely removes the lymph nodes with the highest risk of metastasis, and further axillary treatment may no longer be necessary.

## Data Availability

The datasets used and/or analyzed during the current study are available from the corresponding author on reasonable request. Individual participant data that underlie the results reported in this article, after deidentification (text, tables, figures, and appendices), will be shared. Study protocol will also be available. The data will be available immediately following publication with no end date. Proposals should be directed to chenjiewestchina@163.com; to gain access, data requestors will need to sign a data access agreement.

## Research registration unique identifying number (UIN)

This study was registered with Chictr.org.cn, ChiCTR2300072140.

## Assistance with the study

The authors gratefully acknowledge the whole staff of the Breast Center, West China Hospital, and other centers, who generously helped in the implementation of surgeries and the collection of data throughout the duration of the study

## Presentation

This work was previously presented as a poster at the 19th St. Gallen Breast Cancer Conference (SGBCC), held on March 12–15, 2025, in Vienna, Austria. It was awarded Best Poster and selected for oral poster presentation.
